# Cholangioscopy-guided guidewire insertion into the gallbladder using a novel thin cholangioscope under balloon enteroscopy in a patient with Roux-en-Y gastrectomy

**DOI:** 10.1055/a-2686-2950

**Published:** 2025-09-09

**Authors:** Yuki Tanisaka, Shomei Ryozawa, Masafumi Mizuide, Akashi Fujita, Ryuichi Watanabe, Ryosuke Hamamura

**Affiliations:** 1 183786 Department of Gastroenterology, Saitama Medical University International Medical Center, Hidaka, Japan

Gallbladder drainage is necessary for patients with acute cholecystitis who are unsuitable for surgery. Endoscopic transpapillary gallbladder drainage (ETGBD) is beneficial for patients with coagulopathy and enhances their quality of life due to internal drainage 1 . However, guidewire insertion into the gallbladder through the cystic duct is challenging, as identifying the entrance to the cystic duct is often difficult. To address this, cholangioscopy-guided guidewire insertion is helpful 2 . Recently, it was reported that a novel thin cholangioscope (eyeMAX; Micro-Tech, China), with a length of 219 cm and a diameter of 9 Fr, enables peroral cholangioscopy (POCS)-guided procedures using a balloon enteroscope with a 3.2-mm forceps channel ( Fig. 1 ) 3 4 . We report a case of Roux-en-Y gastrectomy in which POCS-guided guidewire insertion into the gallbladder was successfully performed using a novel thin cholangioscope under balloon enteroscopy.

**Fig. 1:**
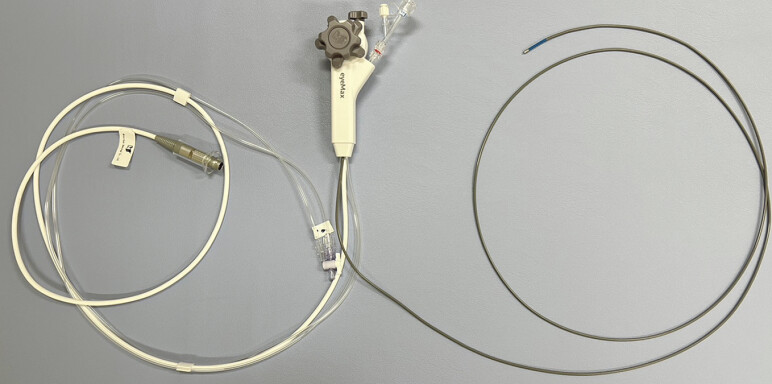
Thin cholangioscope (eyeMAX; Micro-Tech, China) measuring 219 cm in length, with a diameter of 9-Fr.

A 73-year-old man who underwent Roux-en-Y gastrectomy and presented with acute cholecystitis was referred to us ( Fig. 2 ). Since the patient was unsuitable for surgery and had coagulopathy, ETGBD was attempted using a short-type single-balloon enteroscope (SIF-H290; Olympus Marketing, Japan) with a working length of 152 cm and a working channel of 3.2 mm diameter 5 ( Video 1 ). Although fluoroscopy-guided guidewire insertion into the gallbladder was attempted, it was unsuccessful. Subsequently, POCS was performed using a thin cholangioscope. Since the entrance to the cystic duct was identified, POCS-guided guidewire insertion into the gallbladder was successful ( Fig. 3 ). Finally, ETGBD was completed using a 7-Fr plastic stent ( Fig. 4 ).

**Fig. 2:**
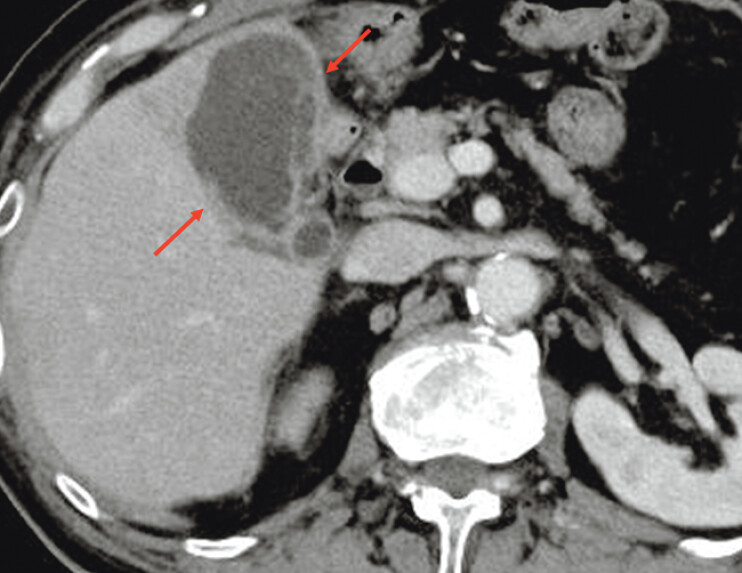
Computed tomography revealing an enlarged gallbladder with wall thickening (red arrow), indicating acute cholecystitis.

**Fig. 3:**
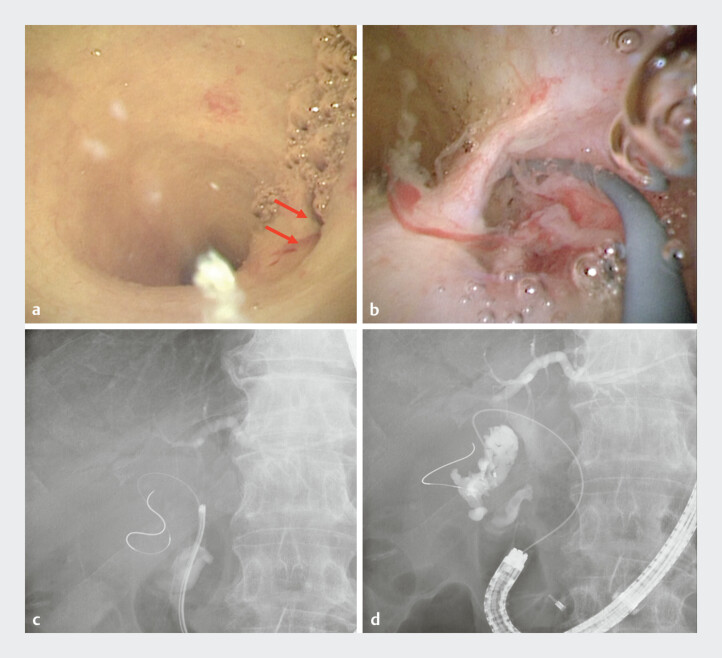
Cholangioscopy and fluoroscopic findings. **a, b** Since the entrance to the cystic duct (red arrow) is identified, the guidewire is inserted under cholangiocopy guidance. **c, d** Fluoroscopy revealing successful guidewire insertion into the gallbladder.

**Fig. 4:**
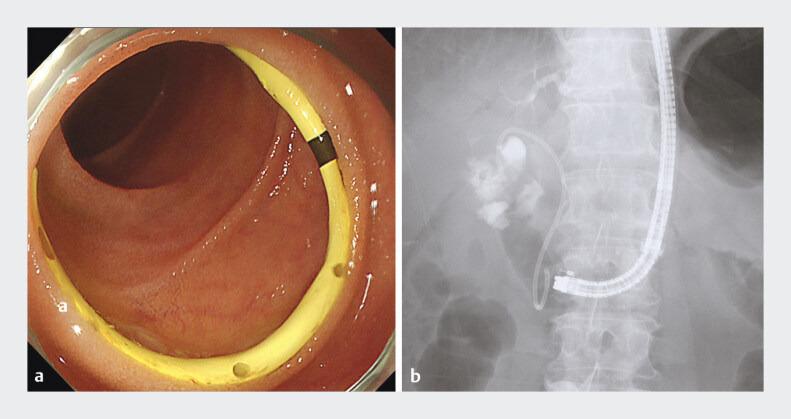
Endoscopic and fluoroscopic findings revealing successful endoscopic transpapillary gallbladder drainage.

Cholangioscopy-guided guidewire insertion into the gallbladder using a novel thin cholangioscope under balloon enteroscopy in a patient with Roux-en-Y gastrectomy.Video 1

Although ETGBD using a balloon enteroscope in patients with Roux-en-Y gastrectomy is considered more challenging compared to those with normal anatomy, this novel thin cholangioscope can be very helpful and improve the success rate of ETGBD in these cases.

Endoscopy_UCTN_Code_TTT_1AR_2AB

## Notes

Endoscopy E-Videos https://eref.thieme.de/e-videos
*E-Videos* is an open access online section of the journal *Endoscopy* , reporting on interesting cases and new techniques in gastroenterological endoscopy. All papers include a high-quality video and are published with a Creative Commons CC-BY license. Endoscopy E-Videos qualify for HINARI discounts and waivers and eligibility is automatically checked during the submission process. We grant 100% waivers to articles whose corresponding authors are based in Group A countries and 50% waivers to those who are based in Group B countries as classified by Research4Life (see: https://www.research4life.org/access/eligibility/ ). This section has its own submission website at https://mc.manuscriptcentral.com/e-videos .
